# Frequency of exposure of endangered Caspian seals to Canine distemper virus, *Leptospira interrogans*, and *Toxoplasma gondii*

**DOI:** 10.1371/journal.pone.0196070

**Published:** 2018-04-26

**Authors:** Somayeh Namroodi, Amir S. Shirazi, Seyyed Reza Khaleghi, James N. Mills, Vahid Kheirabady

**Affiliations:** 1 Department of Environmental sciences, Faculty of veterinary medicine, Gorgan University of Agricultural Sciences & Natural Resources, Gorgan, Golestan Province, Iran; 2 Caspian Seal Treatment and Research Center, Ashooradeh Island, Gorgan, Golestan Province, Iran; 3 Population Biology, Ecology, and Evolution Program, Emory University, Atlanta, Georgia, United States of America; 4 Department of Environment, Gorgan, Golestan Province, Iran; Universita degli Studi di Parma, ITALY

## Abstract

Canine distemper virus (CDV), *Leptospira interrogans*, and *Toxoplasma gondii* are potentially lethal pathogens associated with decline in marine mammal populations. The Caspian Sea is home for the endangered Caspian seal (*Pusa caspica*). In the late 1990s and early 2000s, CDV caused a series of mortality events involving at least several thousand Caspian seals. To assess current infection status in Caspian seals, we surveyed for antibodies to three pathogens with potential to cause mortality in marine mammals. During 2015–2017, we tested serum samples from 36, apparently healthy, Caspian seals, accidentally caught in fishing nets in the Caspian Sea off Northern Iran, for antibodies to CDV, *L*. *interrogans*, and *T*. *gondii*, by virus neutralization, microscopic agglutination, and modified agglutination, respectively. Twelve (33%), 6 (17%), and 30 (83%) samples were positive for CDV, *L*. *interrogans* and *T*. *gondii* antibodies, respectively. The highest titers of CDV, *L*. *interrogans*, and *T*. *gondii* antibodies were 16, 400, and 50, respectively. Frequencies of antibody to these pathogens were higher in seals >1 year old compared to seals <1 year old. Two serovars of *L*. *interrogans* (Pomona and Canicola) were detected. Our results suggest a need for additional studies to clarify the impact of these pathogens on Caspian seal population decline and the improvement of management programs, including systematic screening to detect and protect the remaining population from disease outbreaks.

## Introduction

Caspian seals (*Pusa caspica*), inhabit the frozen areas of the Northern Caspian Sea from late January to the end of April and the southern Caspian Sea shores from May to September. They are the only marine mammal species living in the Caspian Sea [1]. Since the end of the 19^th^ Century, the Caspian seal population has decreased from about one million perhaps a little more than 100,000 [2].

Although the events are poorly documented, at least several thousand Caspian seals died during a series of mortality events due to canine distemper virus (CDV) in the late 1990s and early 2000s [3]. Recently, too few data are available to demonstrate whether Caspian seal populations are continuing to decline. However, due to the relative decline compared to historical abundance, and continued anthropogenic threats such as bycatch in fishing nets and habitat loss, Caspian seals were listed as Endangered in the International Union for the Conservation of Nature Red List of Threatened Species in 2008 [1, 4–6].

Infectious and noninfectious diseases with negative effects on longevity or reproductive capacity can severely impact seal populations, especially when anthropogenic disturbances cause immunosuppression [7]. Pathogens that are major causes of mortality in marine mammals include: morbilliviruses (e.g., CDV), herpesviruses, influenza A virus, adenoviruses, caliciviruses, *Leptospira* spp., *Brucella* spp., and *Toxoplasma gondii* [8–10]. Despite recognized mass mortalities caused by CDV, knowledge of the impacts of other potentially lethal pathogens on Caspian seals is very limited and the current status of CDV infection in Caspian seals is unclear [3].

*Leptospira interrogans* is a bacterial species consisting of a wide variety of serovars that cause leptospirosis in warm-blooded animals nearly worldwide. The great diversity of serovars combined with the characteristic that the bacteria are excreted in the urine of infected animals for long periods, means that there are many sources of *L*. *interrogans*, which have long survival times in warm-moist environments with neutral pH. Direct transmission may occur through contact with contaminated urine and indirect transmission from contaminated food, water, or other substances. The primary routes of entry of the bacteria into the host body are through broken skin, the conjunctiva, or through the placenta [11]. The *Leptospira* microscopic agglutination test (LMAT) is a well-established standard test for diagnosing leptospirosis in many species of animals including some marine mammals such as the California sea lion (*Zalophus californianus*) [12]. Despite deadly outbreaks of leptospirosis in California sea lions and northern fur seals (*Callorhinus ursinus*), the signs of leptospirosis are not well known in most marine mammal species [13, 14]. Depending on the immune system status of the host, interstitial nephritis, dehydration, polydipsia, vomiting, depression, and abortion can be typical signs of infection in marine mammals [14].

The zoonotic intracellular parasite, *T*. *gondii*, can infect probably any warm-blooded animal, but felids are the definitive hosts. Toxoplasmosis, can be deadly in immunosuppressed hosts [15]. Infection with *T*. *gondii* in marine mammals can cause neonatal death [16]. It is also an important cause of mortality in sea otters (*Enhydra lutris nereis*) with encephalitis and it causes fatal toxoplasmosis in Pacific harbor seals (*Phoca vitulina richardsi*) [10, 17]. Sporadic cases of lethal toxoplasmosis in marine mammals are frequently associated with immunosuppression resulting from morbillivirus infection [10]. *T*. *gondii* infection may indirectly lead to death of marine mammals by affecting their behavior and increasing the risk of trauma and death by hunters [17].

Transboundary migration of Caspian seals through the waters of the countries bordering the Caspian Sea and the variability of migratory activities among individuals [18], combined with the inadequate implementation of a systematic plan for recovery and maintenance of the species, have hindered research into the diseases of Caspian seals. The status of infection or disease in Caspian seals associated with the three important pathogens described above is completely unknown. Baseline data about the susceptibility to, and frequency of these pathogens in these endangered seals is critical to improving captive care and recovery efforts of wild populations. We used antibody screening to measure the frequency of exposure of Caspian seals on the Caspian Sea coast of Northern Iran to CDV, *L*. *interrogans*, and *T*. *gondii*.

## Materials and methods

### Ethics statement

Our research protocol was approved by the research and ethics committee of the Deputy of Natural Environment of Golestan Province, Department of Environment (S1 Certificate, permit number: 125/7894).

### Sample collection and clinical examination

Caspian seals accidentally caught in fishing nets by fishermen near the coast of Northern Iran were physically restrained and transported to Seal Treatment and Research Center (Ashooradeh Island, Golestan Province) by suitable vehicle during 2015–2017 [19]. Animals were handled in strict compliance with the European Union legislation on the protection of animals used for scientific purposes (EU Directive 2010/63/EU) [20].

In the rehabilitation center, seals were housed individually in a pool of fresh saltwater from the Caspian Sea and provided with food, water, and any necessary medicines. Animal welfare was monitored daily. After seals were treated and allowed to recover from any injuries and from the stress of capture, blood collection and flipper tagging were performed under physical restraint as described by Lynch and Bodley [19].

Sex, site of sampling, and clinical signs were recorded. Seals were assigned to one of two age classes. The youngest seals captured were less than one year old, but older than 3–6 months (because the first 3–6 months of life are spent in the Northern Caspian Sea) [1]. We refer to this age class as “yearlings.” The “older” age class consisted of those seals greater than 1 year old. The distinction between age classes was made on the basis of size, pelage, and appearance of teeth. Blood samples were collected from the caudal gluteal vein using Venoject samplers (Novingostar Co., Tehran, Iran). Blood samples were allowed to clot and centrifuged for 10 min at 3000 rpm. Sera were removed and stored at -20°C. The period of rehabilitation varied from 1 to 4 weeks, but averaged about 3 weeks. Once rehabilitation was complete, flipper tagging was performed using sheep ear tags (Part Imensill Co., Tehran, Iran) and animals were released on the shore near the location they were captured.

### CDV antibody detection

We detected CDV antibody using the microplate virus neutralization test (VNT) as described by Saliki and Lehenbauer [21]. The Onderstepoort vaccine strain of CDV was used as antigen. After dilution of sera starting from 1:2, plates were incubated at 37°C in 5% C0_2_ for 3–5 days. A dilution of 1:8 was considered the VNT-positive cut-off point.

### *L*. *interrogans* antibody detection

Sera were examined for six *L*. *interrogans* serotypes: Pomona, Grippotyphosa, Icterohaemorrhagiae, Canicola, Hardjo, and Australis, by *Leptospira* microscopic agglutination test (LMAT), in the Leptospira Research Laboratory, Faculty of Veterinary Medicine, University of Tehran. The LMAT was run on sera in a dilution series from 1:100 to 1:2800. Positive and negative standard samples were added to two wells of each micro-titration plate and the plates were incubated at 30°C for 2 hr. The serum sample was considered positive for antibody to a *L*. *interrogans* serovar if there was ≥ 50% *Leptospira* agglutination at a serum dilution of ≥ 1:100 when observed by dark-field microscopy [12].

### *T*. *gondii* antibody detection

To detect *T*. *gondii* antibody, we used the *Toxoplasma* modified agglutination test (TMAT) as validated by Dubey (1997). *T*. *gondii* tachyzoites were suspended in mercaptoethanol, and then this suspension was used to dilute serum samples in a 2-fold series from 1:25 to 1:500 [22]. There are no data on the specificity and sensitivity of this test, nor is there an accurate cut-off titer for Caspian seals. So, according to previous surveys by Aguirre et. al., ≥ 1:25 dilution (Titer ≥ 25) was considered *T*. *gondii* antibody positive [23].

### Statistical analysis

Results were entered into SPSS (v20) software and Chi square tests were used to determine the significance of differences in antibody frequencies between age classes.

## Results

All 36 Caspian seals tested during the study appeared healthy on clinical examination. Seventeen seals were male and 19 were female; 16 were yearlings (< 1 year old) and 20 were older. Of the 36 serum samples, 12 (33%), 6 (17%), and 30 (83%) were antibody-positive to CDV, *L*. *interrogans*, and *T*. *gondii*, respectively. The highest titers to CDV, *L*. *interrogans*, and *T*. *gondii* were 16, 400, and 50 respectively. Two serovars of *L*. *interrogans* (Pomona and Canicola) were detected in six serum samples (S1 Table). There were statistically significant differences in frequencies of antibody between yearling and older seals and there were lower antibody frequencies in yearlings than in older seals (CDV: X^2^ = 5.625, df = 1, p = 0.018, *L*. *interrogans*: X^2^ = 5.760, df = 1, p = 0.016, *T*. *gondii*: X^2^ = 9, df = 1, p = 0.003). Each positive serum had detectable antibody to only one serovar of *L*. *interrogans* (Table 1).

**Table 1:** Frequencies and antibody titers detected in sampled Caspian seals by age class.

Pathogen	Pos^a^/tested	Pos/serovar/titer^b^	Yearlings Pos/tested/(%)	Older Pos/tested/(%)
CDV	12/36	6/8 6/16	2/16(12%)	10/20(50%)
*L*. *interrogans*	6/36	3/Pomona/200 1/Pomona/400 1/ Canicola /100 1/ Canicola /200	0/16(0%)	6/20(30%)
*T*. *gondii*	30/36	24/25 6/50	10/16(62%)	20/20(100%)

^a^Pos = Positive

^b^Serovars provided only for *L*. *interrogans*; titer expressed as reciprocal of highest dilution with positive result.

## Discussion

### Exposure to CDV

Since the late 1980s there has been much evidence of mass mortality in marine mammals due to morbillivirus infection [3, 24]. These viruses have been detected in cetaceans from the North Pacific and many species of marine mammals from the Atlantic Ocean, Mediterranean Sea, and Arctic waters, leading to at least eight epizootics [25–27]. The literature suggests that morbillivirus infection has played a much deadlier role in marine mammal populations than toxoplasmosis or leptospirosis.

CDV has been diagnosed in mass mortalities of Baikal seals and Caspian seals, and there are a few reports of CDV antibody in other marine mammal species throughout the world [3, 28]. Antibody to CDV was detected in 32 of 96 sampled crabeater seals (*Lobodon carcinophagus*) and two of three leopard seals (*Hydrurga leptonyx*) from Antarctica [29], and in 12 of 40 harp seals (*Pagophilus groenlandicus*) from Greenland [29].

Our data indicate that CDV is still present in the Caspian seal population and that it is not 100% lethal. The moderate frequency of CDV antibody in Caspian seals and the currently low population size argue against sustained, long-term, endemic infection in the population. Although Kuiken et al. (2006) suggested that the Caspian seal population itself could be acting as a reservoir for CDV, they based their calculations on a seal population size of 360,000–400,000, and an estimate that a population size of 120,000 was sufficient to maintain infection [30]. The currently estimated population size of 104,000 to 168,000 makes endemic maintenance less likely [5], but the population may still be above the estimated 120,000 necessary to maintain endemic infection. An alternative source of CDV for Caspian seals may be persistent infection in terrestrial carnivores along the Caspian Sea coast [3]. In the end, it is still impossible, at this time, to definitively implicate either endemic infection or spillover.

### Exposure to *L*. *interrogans*

The 17% antibody frequency in our sample of apparently healthy Caspian seals suggests that seals are commonly exposed to *L*. *interrogans* and that the infection is (at least) not 100% fatal. Antibody frequency may actually be higher than 17%, as the LMAT has not been certified for antibody detection in Caspian seals and sensitivity may be less than 100%. It is also possible that antibody may become undetectable over time. Finally, LMAT may not detect some positive animals due to transient nature of bacteria and bacteria may be hiding in the kidney and genital tracts of infected animals [15].

*L*. *interrogans* serovars Pomona and Canicola have been reported to infect other species of marine mammals. Antibody to serovars Canicola and Pomona were detected in New Zealand fur seals (*Arctocephalus forsteri*) [7] and antibody to serovars Hardjo, Bratislava, Grippotyphosa, Cynopteri, Bataviae, Ichterhaemorrhagiae, and Pyrogenes have been described in other marine mammals [7, 31].

Some serologic studies on marine mammals using LMAT showed reaction to more than one serovar. These results could have resulted from mixed infections or from cross reaction among serovars on the LMAT [31]. However, in our study, all positive sera were reactive with only one serovar.

*Leptospira* serovar dominance and antibody frequency in susceptible animals can vary based on geographic area, susceptible species of terrestrial animals in each region, number of animals tested, and the test used [15]. Because the common serovars in a given region may vary over time, frequent screening and identification of serovars in each region has been suggested to maintain effective control programs [32].

Because leptospires cannot survive in sea water, detection of *Leptospira* antibody in Caspian seals suggests that the seals might have been in close contact with other infected animal species on rookeries. Transfer of maternal antibody to pups may occur, however [15], and may or may not be protective against infection. Finally, although leptospires do not survive in “sea water”, the salinity of the Caspian Sea water is far lower (about one third) than the earth’s oceans, varying between 1 part per thousand and about 13 parts per thousand. The lowest values are in the northern basin where the Volga and Ural rivers discharge [33]. To our knowledge, it is not known how this low salinity might alter the survival of leptospires, if at all.

Dogs are a common maintenance host of *L*. *interrogans* serovar Canicola, and cattle, sheep, and pigs are common maintenance hosts of serovar Pomona [34]. So, these domestic animal species could be sources of exposure to Caspian seals, especially in Northern Iran, where seals are seen in the near-shore waters and even hauled-out near small villages where inhabitants keep ruminants. These shoreline areas are heavily polluted and contaminated by human refuse, roaming dogs and cats, and occasionally free livestock. Currently, these reports are only anecdotal and specific studies are needed. In more remote areas, rodents or wild canids such as wolves or jackals are another possible source. Serovars Canicola and Pomona are now the most common *L*. *interrogans* serovars in some parts of Northern Iran [32]. It is also possible that *L*. *interrogans* serovars could be maintained within the seal population and exposure to contaminated seal urine/feces on haul-out sites would be a plausible route of intraspecific transmission.

The migratory lifestyle of Caspian seals makes the *Leptospira* sources and the locations of transmission sites difficult to determine [18]. However, vaccination of susceptible domestic animals, when feasible, and restriction of contact between Caspian seals and other animals at haul-out sites would likely prevent transmission of *Leptospira* to seals. Since, *Leptospira* shedding in urine samples has not been investigated in Caspian seals, the possible role of seemingly healthy seals in bacterial shedding remains unclear. Additional studies are needed to determine if Caspian seals can be maintenance hosts for *L*. *interrogans* serovars Canicola and Pomona. Nevertheless, humans in contact with Caspian seals should be aware of the potential risk of *Leptospira* shedding.

Our results provide basic data about the epidemiology of *L*. *interrogans* infection in Caspian seals. Because other species of marine mammals have suffered mass die-offs due to *Leptospira* infection, understanding the consequences of *Leptospira* infection in Caspian seal individuals is critical to understand the possible impact of infection on their populations [31].

### Exposure to *T*. *gondii*

The high frequency of *T*. *gondii* antibody in sampled Caspian seals can be explained by contamination of the marine environment by freshwater runoff containing oocysts from felids and the dissemination of these oocysts by currents. These oocysts can then become sporulated and may remain infectious in seawater for as much as two years [35]. Similarly, Alvarado-Esquivel et al. [36] reported *T*. *gondii* infection in 87% of captive Atlantic bottlenose dolphins (*Tursiops truncates gillii*) in Mexico. Conversely, Aguirre [23] reported a low prevalence of *T*. *gondii* antibody in Hawaiian monk seals (*Monachus schauinslandi*) in the northwestern Hawaiian Islands. Bearded seals (*Erignathus barbatus*), walruses (*Odobenus rosmarus)*, California sea lions (*Zalophus californianus*), ringed seals (*Phoca hispida*), and spotted seals (*Phoca largha*) have also shown evidence of *T*. *gondii* infection [37].

Despite the absence of Caspian seals with clinical signs of toxoplasmosis in this study, the high frequency of *T*. *gondii* antibody raises suspicion that *T*. *gondii* infection may be, or may become, a health problem for this species, especially in immunosuppressed individuals [10]. After all, seriously diseased animals might quickly be removed from the sampling pool leading to findings of lower *T*. *gondii* antibody frequencies in sampled seals. Management directed at minimizing the exposure of Caspian seals to *T*. *gondii* oocysts, including restriction of seal contact with felids would help prevent infection.

Frequencies of exposure to all three pathogens were lower in yearlings than older Caspian seals. This is expected and is reflective of the very limited opportunities for exposure for seals during the first year of life, half of which is spent in the northern Caspian Sea. It may be possible however that some infected yearlings may have died and thus were eliminated from the sampling population. Results of some studies have differed. For example, Alvarado-Esquivel [36] reported no significant variation in *T*. *gondii* antibody prevalence among age classes of marine mammals. Nielsen et al. [38] found that PDV antibody prevalences in Atlantic walrus (*Odobenus rosmarus rosmarus*) were not associated with sex or age and that CDV antibody prevalences increased with age in crabeater and leopard seals. Colagross-Schouten et al. [12] reported greater *L*. *interrogans* serovar Pomona exposure in adult and subadult sea lions than in juveniles.

Without much more extensive sampling, and an understanding of any pathogenic effects of these pathogens on Caspian seals, it is impossible to predict the epizootic or endemic infection of Caspian seals by these three pathogens, or the effects of such infection on the population. The circulation of enzootic, host-adapted *Leptospira* serovars in California sea lions, for example, has been cited to explain the high serum antibody prevalence, low antibody titers, and absence of clinical signs of leptospirosis in pups [11]. In our case, the low titers for CDV (8–16), *T*. *gondii* (25–50) and *L*. *interrogans* (100–400) in apparently healthy Caspian seals could possibly indicate chronic exposure to these agents or simply decreasing titers over time following previous exposure. Sample handling and an imperfect cold chain could also have contributed to the low titers observed.

Climatic conditions along the coasts of countries bordering the Caspian Sea are mostly appropriate for the survival of the three pathogens we studied. For example, 35% of rice farms and water canals, and 30% of rivers in Gilan Province, Iran, which borders the Caspian Sea, are contaminated with *Leptospira* spp. [39]. However, seasonal movement of Caspian seals shows the need for molecular and phylogenetic analyses on isolated pathogens in all countries bordering the Caspian Sea to detect potential sources and regions of CDV, *T*. *gondii*, and *L*. *interrogans* exposure to Caspian seals.

Our results provide basic data about the natural threats that may impact the Caspian seal population. However, understanding the importance of antibody-positive animals without clinical signs or isolation of the pathogens begs the important question of whether *T*. *gondii* and *Leptospira* can lead to deadly disease. Because Caspian seals are highly susceptible to CDV infection, emphasis should be placed on minimizing the risk of a new epidemic. On the basis of our data we cannot predict if CDV will cause future outbreaks in Caspian seals. Systematic screening for CDV (especially), *L*. *interrogans*, and *T*. *gondii* in surviving Caspian seals using molecular techniques is also needed.

Although a Caspian Seal Conservation Action Plan developed by the United Nations Development Program, Caspian Environment Program (http://projects.inweh.unu.edu/inweh/display.php?ID=1057)) recommends “periodic health surveys” for Caspian seals, the plan lacks specifics. It does not identify potentially important diseases, and has not been fully implemented. We strongly encourage the careful planning and vigorous implementation of a plan that would establish long-term monitoring of the general health of Caspian seals in the remaining population, identify the sources of CDV, *L*. *interrogans*, *T*. *gondii*, and other pathogens affecting Caspian seals, and take steps to eliminate or reduce exposure of seals to these pathogens.

## Supporting information

S1 Certificate Approval certificate for Caspian seal project study. (DOCX)

S1 Table Antibody titers for sampled Caspian seals by age class, sex, and year of sampling. (DOCX)
