# Supplementary material for: Frequency of exposure of endangered Caspian seals to Canine distemper virus, Leptospira interrogans, and Toxoplasma gondii
Source: PLoS One. 2018 Apr 26;13(4):e0196070. doi: 10.1371/journal.pone.0196070 (PMC5919510; doi:10.1371/journal.pone.0196070)

This official letter was prepared because of our application on a formal report about the animal ethics

The following is an English translation of the text of the following document:

Certificate of Approval for Caspian Seal Project Study

I hereby certify that. Dr. Amir Sayyad Shirazi, veterinarian and member of the Caspian Seal Treatment and Research Center, has been working with the Golestan Province Department of Environment as a supervisor of the project entitled: “Preparation of a report on the situation

(health status) of the Caspian seal”, under contract number: 125/7894 approved on: 2015/3/19.

The activities (surveys) of Dr. Amir Sayyad Shirazi on Caspian seals are being conducted in accordance with laws and rules of ethics of working with wildlife (animal ethics) of the Iran Department of Environment and are under the supervision of this department.

On behalf of Mojtaba Hoseini, Deputy Minister of Natural Environment

Esmail Mohajer, General Manager of Golestan Province Department of Environment (who signed the letter)


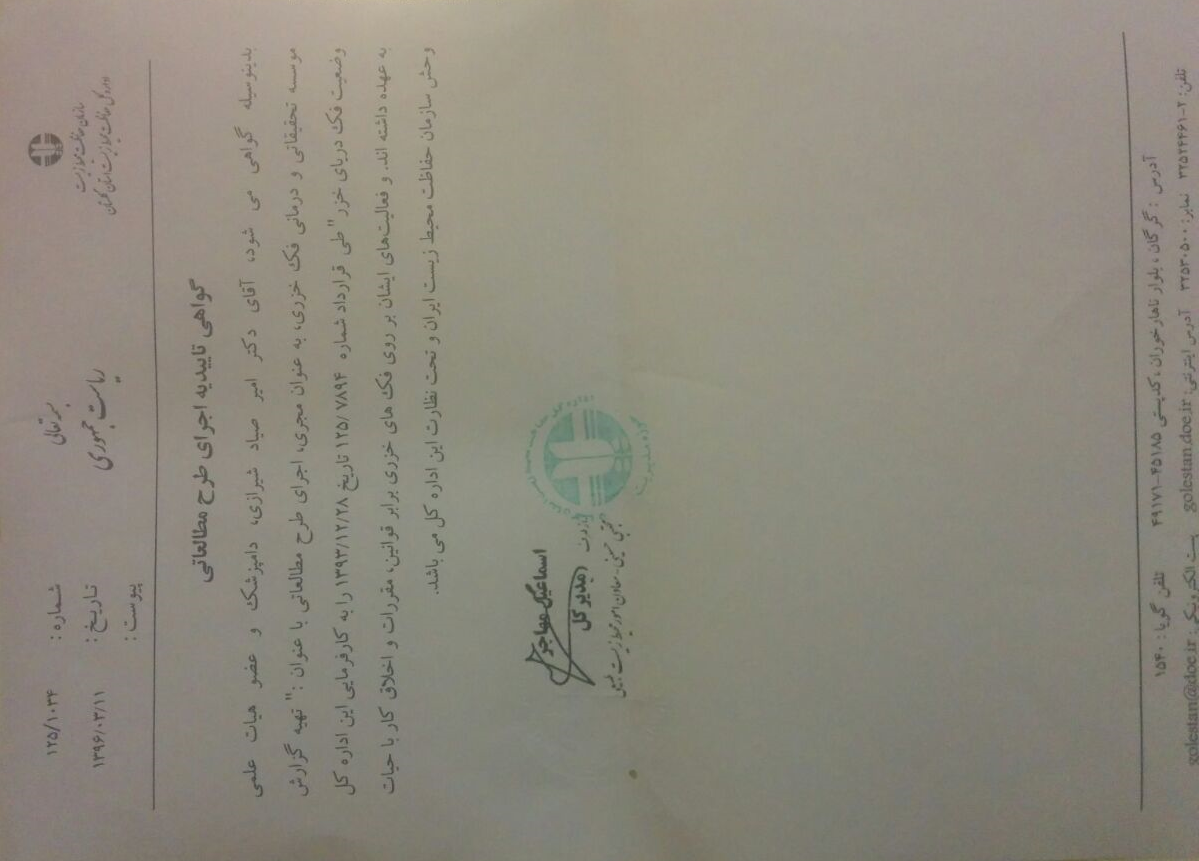

Supplement: S1 Certificate — (DOCX) [file pone.0196070.s001.docx]
