# Supplementary material for: Frequency of exposure of endangered Caspian seals to Canine distemper virus, Leptospira interrogans, and Toxoplasma gondii
Source: PLoS One. 2018 Apr 26;13(4):e0196070. doi: 10.1371/journal.pone.0196070 (PMC5919510; doi:10.1371/journal.pone.0196070)
Supplement: S1 Table — (DOCX) [file pone.0196070.s002.docx]

Supporting information 1

**Frequency of exposure of endangered Caspian seals to Canine distemper virus, *Leptospira* *interrogans,* and *Toxoplasma gondii***

Somayeh Namroodi^1,*^, Amir S, Shirazi^2^, Seyyed Reza Khaleghi^1^, James N. Mills^3^, Vahid Kheirabady^4^

S1, Table A: Antibody titers detected in sampled Caspian seals by age class, sex, and year of sampling.

| Seal ID Number | Sex | *T. gondii* antibody titer | CDV antibody titer | *L. interrogans* (Canicola) antibody titer | *L. interrogans* (Pomona) antibody titer | Year of sampling | Age group |
| --- | --- | --- | --- | --- | --- | --- | --- |
| 1 | F | 50 | N | N | N | 2015 | Y |
| 2 | F | N | N | N | N | 2015 | Y |
| 3 | M | 25 | 8 | N | N | 2015 | Y |
| 4 | F | 25 | N | N | N | 2015 | Y |
| 5 | M | N | N | N | N | 2015 | Y |
| 6 | F | 25 | N | N | N | 2015 | Y |
| 7 | M | 25 | N | N | N | 2015 | Y |
| 8 | M | N | N | N | N | 2016 | Y |
| 9 | M | 25 | N | N | N | 2016 | Y |
| 10 | F | N | N | N | N | 2016 | Y |
| 11 | M | N | N | N | N | 2016 | Y |
| 12 | F | 25 | 16 | N | N | 2016 | Y |
| 13 | M | 25 | N | N | N | 2016 | Y |
| 14 | M | 25 | N | N | N | 2016 | Y |
| 15 | F | N | N | N | N | 2016 | Y |
| 16 | M | 50 | N | N | N | 2016 | Y |
| 17 | F | 50 | 16 | N | N | 2015 | O |
| 18 | M | 25 | 8 | N | N | 2015 | O |
| 19 | F | 25 | N | N | 200 | 2015 | O |
| 20 | M | 25 | 16 | N | N | 2015 | O |
| 21 | F | 50 | N | 100 | N | 2015 | O |
| 22 | F | 25 | 8 | N | 200 | 2015 | O |
| 23 | F | 25 | N | N | N | 2015 | O |
| 24 | M | 25 | 8 | N | N | 2015 | O |
| 25 | F | 25 | N | N | N | 2015 | O |
| 26 | M | 25 | 16 | N | N | 2015 | O |
| 27 | F | 25 | 8 | N | N | 2015 | O |
| 28 | F | 25 | N | N | N | 2016 | O |
| 29 | F | 25 | N | N | N | 2016 | O |
| 30 | F | 25 | N | N | N | 2016 | O |
| 31 | F | 25 | 16 | N | N | 2016 | O |
| 32 | M | 50 | N | N | 200 | 2016 | O |
| 33 | M | 25 | N | N | 400 | 2016 | O |
| 34 | M | 25 | 8 | N | N | 2016 | O |
| 35 | M | 25 | N | 200 | N | 2016 | O |
| 36 | F | 50 | 16 | N | N | 2016 | O |

Y = Yearling, O = Older, F = Female, M = Male, N = Negative
